# Association between GLP-1 receptor agonists as a class and colorectal cancer risk: a meta-analysis of retrospective cohort studies

**DOI:** 10.1186/s12876-025-04211-4

**Published:** 2025-08-22

**Authors:** Ying Zhong, Tingting Wu, Najeeb Ullah Khan

**Affiliations:** 1 Traditional Chinese Medicine Proctology Department, West China Hospital of Sichuan University-Ziyang Hospital (Ziyang Central Hospital), Ziyang City, 641300 China; 2 Department of Gastroenterology, West China Hospital of Sichuan University-Ziyang Hospital, Ziyang Central Hospital, Ziyang City, 641300 China; 3 Institute of Biotechnology & Genetic Engineering (Health Division), The University of Agriculture Peshawar, Peshawar, 25130 Pakistan

**Keywords:** GLP-1 receptor agonists, Type 2 diabetes mellitus, Colorectal cancer, Incidence of CRC, Meta-analysis

## Abstract

**Background:**

Glucagon-like peptide-1 receptor agonists (GLP-1 RAs) are extensively used in the management of type 2 diabetes mellitus (T2DM) and obesity. While these medications offer glycemic control and cardiovascular benefits, the risks have increased because of their potential impact on cancer risk, particularly colorectal cancer (CRC). This meta-analysis aimed to evaluate the association between GLP-1 RAs and CRC risk in patients receiving GLP-1 RAs.

**Methods:**

This study was conducted the PRISMA guidelines. Electronic databases (PubMed, Embase, Cochrane Library Web of Science, and ClinicalTrials.gov) were searched from inception to December 2024. The inclusion criteria encompassed Studies analyzing the effects of GLP-1 RA on CRC risk in patients with T2DM. The Newcastle-Ottawa Scale was used for the quality assessment of the included cohort studies. Random-effects models were employed for the pooled analysis, and heterogeneity was evaluated using the I2 statistic.

**Results:**

Seven retrospective cohort studies involving 5,066,681 patients were included. The pooled analysis revealed a significantly increased risk of CRC among patients receiving GLP-1 RAs (RR, 2.31; 95% CI, 1.82–2.93; I2 = 36%; *p* < 0.0001). However, the incidence of CRC was not significantly associated with GLP-1 RA use compared with other drugs (OR, 1.73; 95% CI: 0.21–14.18, *p* = 0.61; I2 = 100%). Quality assessment indicated a low-to-moderate risk of bias across the included studies.

**Conclusion:**

Overall, this study suggests a significantly increased risk of colorectal cancer associated with GLP-1 RA use in patients receiving GLP-1 RAs. However, the incidence of CRC is not considerably high. These findings highlight the need for further long-term, large-scale clinical trials to elucidate the relationship between GLP-1 RAs and cancer risk. Clinicians should consider these results when prescribing GLP-1 RAs, particularly in patients with CRC risk factors.

**Graphical Abstract:**

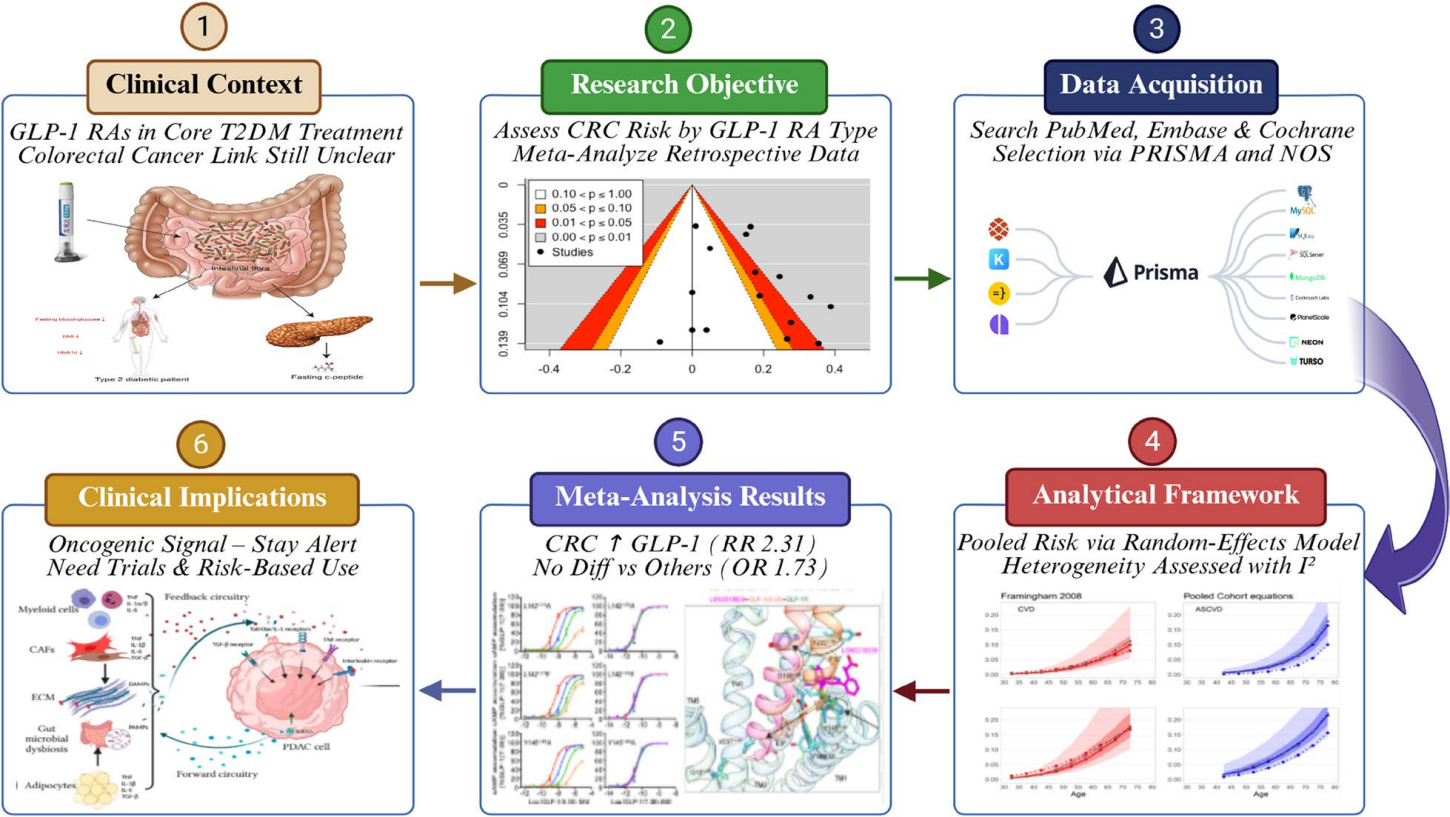

**Supplementary Information:**

The online version contains supplementary material available at 10.1186/s12876-025-04211-4.

## Background

Glucagon-like peptide-1 receptor agonists (GLP-1 RAs) are extensively used in the management of patients with type 2 diabetes mellitus (T2DM) and obesity [1]. These agents also maintain weight and cardiovascular health, along with glycemic control, to reduce long-term complications and improve the quality of life among patients with T2DM [2, 3]. GLP-1 RAs have functions similar to gut hormones, endogenous GLP-1, and different GLP-1 drugs include albiglutide, exenatide, efpeglenatide, orforglipron, dulaglutide, lixisenatide, liraglutide, and semaglutide [4]. Pancreatic beta cells respond to GLP-1 agents by activating GLP-1 receptors that inhibit glucagon release, secrete glucose-dependent insulin, promote satiety, and slow gastric emptying through multiple metabolic pathways [5, 6]. Additionally, new GLP-1 agonists, such as cotadutide and tirzepatide, act on multiple receptors, including GLP-1, to provide more effective metabolic control.

Despite their benefits, GLP-1 RAs, are associated with several adverse effects, including nausea, diarrhea, vomiting, constipation, gastroparesis, pancreatitis, dyspepsia, abdominal pain, and gallbladder disease [7, 8]. Some studies have also raised concerns about their potential to increase the risk of gastrointestinal and colorectal cancers [9, 10].

Colorectal cancer (CRC) remains one of the leading causes of cancer-related morbidity and mortality globally. Overall, total cases of CRC account for 9.4% of cancer-linked mortalities and 10% of newly diagnosed cancer cases [11, 12]. The epidemiological data suggested that CRC is the third most commonly diagnosed cancer in men and second in women, with a leading cause of cancer-related deaths [13, 14]. In 2020, an estimated 1.9 million new cancer cases and almost 935,000 cancer deaths were reported worldwide, with an incidence of 11% of all cancer diagnoses [13]. The GLOBOCAN 2020 stated that the occurrence and mortality access to healthcare. Factors that influence the increasing incidence of cancer-related disorders include health-related behaviors and social aspects. It is expected that the CRC will increase by 60% to over 2.2 million new cases and 1.1 million deaths by 2030 [15].

The global burden of type 2 diabetes mellitus (T2DM) is escalating at an alarming rate, with an estimated 537 million adults affected worldwide in 2021—a figure projected to rise to 783 million by 2045, according to the International Diabetes Federation [16]. Population aging, sedentary lifestyles, unhealthy diets, and urbanization primarily drive this surge [17, 18]. As a result, the demand for effective long-term glycemic control has led to the widespread use of glucagon-like peptide-1 receptor agonists (GLP-1 RAs), which have become a cornerstone in the management of T2DM [19–21]. However, growing clinical attention is being directed toward the potential long-term effects of individual GLP-1 RAs, particularly their association with cancer risks such as colorectal and pancreatic cancers [22]. Understanding these differential effects is critical, mainly as these agents are increasingly prescribed to high-risk populations. Identifying GLP-1 RAs with protective or adverse impact on cancer risk could significantly inform therapeutic decisions, cancer screening protocols, and long-term patient outcomes [17, 18]. Therefore, a thorough understanding of cancer and its modifiable risk factors is essential for developing effective early detection and prevention strategies.

Obesity, diabetes, and cancer risks have complex and multifaceted interactions. In recent years, the potential extra glycemic effects of GLP-1 RAs have been observed in the form of increased risk of cancer [18]. Previous cohort-based studies have reported a reduced risk of cancers such as colorectal cancer [23], pancreatic cancer [24], and hepatocellular carcinoma [25] after treatment with GLP-1 RAs. GLP-1 receptor signaling has been reported to control mucosal expansion in the intestine and colon, thus playing a role in intestinal tumorigenesis [26]. Epidemiological research has linked obesity and T2DM to increased risk of colorectal cancer and other malignancies [27]. Several hypothesized pathways could lead to an elevated risk of cancer, including insulin resistance, hyperinsulinemia, chronic inflammation, and altered gut flora. Scientists are interested in how GLP-1 receptor agonists affect colorectal carcinogenesis because they directly alter gut physiology by slowing stomach emptying, increasing gut hormone release, and altering microbiota composition [28].

However, inherent biases and confounding factors, such as heterogeneity in patient demographics, study designs, and the presence of additional cancer risk factors, make it challenging to interpret the results of observational studies. A comprehensive and methodical review is may be attributed to variations in study techniques, follow-up periods, or patient demographics. Understanding the safety profile of GLP-1 RAs, particularly regarding the risk of colorectal cancer, is crucial given their extensive and expanding use. Thus, this study aimed to evaluate the potential association between GLP-1 receptor agonists and colorectal cancer (CRC) risk among patients by adopting a systematic review and meta-analysis approach.

## Methods

This meta-analysis was performed by following the “Reporting Items for Systematic Review and Meta-Analysis (PRISMA)” guidelines [29] to fulfill research aims. There was no need for further ethical review, as the inclusion of previously published retrospective and prospective cohort studies was deemed sufficient.

### PICO framework

This study used the Population Intervention Control Outcome Study design (PICOS) framework to guide the search [30].

#### P (Population)

Adults (typically with type 2 diabetes or obesity) receiving pharmacologic treatment.

### I (Intervention): treatment with GLP-1 receptor agonists

#### C (Comparison)

 Patients not receiving GLP-1 receptor agonists (e.g., receiving other antidiabetic agents or no GLP-1 RA exposure).

#### O (Outcome)

The primary outcomes were colorectal cancer risk (Hazard Ratio) and incidence of colorectal cancer.

#### S (Study design)

Prospective and retrospective cohort studies.

### Search straegy

The PRISMA guidelines aided in the selection of research papers relevant to the study’s aims. Research articles were searched using three electronic databases: PubMed, Embase, the Cochrane Library, Web of Science, and ClinicalTrials.gov from inception to December 2024. The MesH keywords for data search from electronic databases were (“glucagon-like peptide-1 receptor agonists’ OR “GLP-1 RAs’ OR “glycemic control”) AND (“colorectal cancer risk” OR “CRC” OR “colon risk’ OR “colorectal neoplasms’ OR “colorectal cancer risk”). We carefully reviewed the reference lists of all previous systematic reviews and meta-analyses to identify additional research articles. We also searched for grey literature, conference abstracts.

### Eligibility criteria

The eligibility criteria were used to select and screen research studies after searching the electronic databases.

### Inclusion criteria

1. Studies analyzed the patient population receiving GLP-1 RAs (having age > 18 years).
2. Studies analyzed the effects of GLP-1 RAs on colorectal cancer risk.
3. Studies tracking patient outcomes related to colorectal cancer risk and incidence rates of colorectal cancer.
4. Primary research studies, such as prospective and retrospective cohort studies.
5. Studies published in the English language and those with full text available.

### Exclusion criteria

Those studies were excluded:

1. Studies analyzing the effects of other drugs such as DPP-4.
2. Studies based on systematic reviews, meta-analyses, comprehensive reviews, narrative reviews, and editorials.
3. Studies with non-full-text papers published in languages other than English.

### Data extraction

Two independent reviewers extracted the data to be placed in a prespecified table. The studies obtained from the database search were entered into the EndNote library. A librarian performed the searches. Duplicates were excluded from the next step. The reviewers applied the eligibility criteria in a blinded manner to all the individual studies. Discrepancies were sorted by mutual agreement among authors. The third reviewer was involved to resolve disparities. Data related to demographic information, such as authors, year of study, country, study population, study design, and primary outcomes, such as colorectal cancer risk, were extracted (Table 1).

### Quality assessment

The quality of the included studies was evaluated by the research design using appropriate instruments. The Newcastle-Ottawa Scale (NOS) was used for quality assessment, as observational studies were included [20]. Included studies were classified as low-risk if their score was greater than 7, moderate-risk if their score was between 5 and 7, and high-risk if their score was less than 5. Consensus was used to settle any disputes regarding risk bias assessment [31].

### Statistical analysis

Statistical analyses were performed using Review Manager Software (Cochrane Collaboration, version 5.4.0). Random-effects models were used for the pooled data analysis of studies with potential heterogeneity. Statistical significance was set at *P* < 0.05. The I2 statistic assessed heterogeneity, and considerable heterogeneity was indicated by I2 values > 50%. The results of this analysis were combined, including the risk and incidence [32]. If sufficient granular data is reported among the included studies, then subgroup analysis based on individual GLP-1 RAs can be performed.

## Results

### Search results

**Fig. 1:**
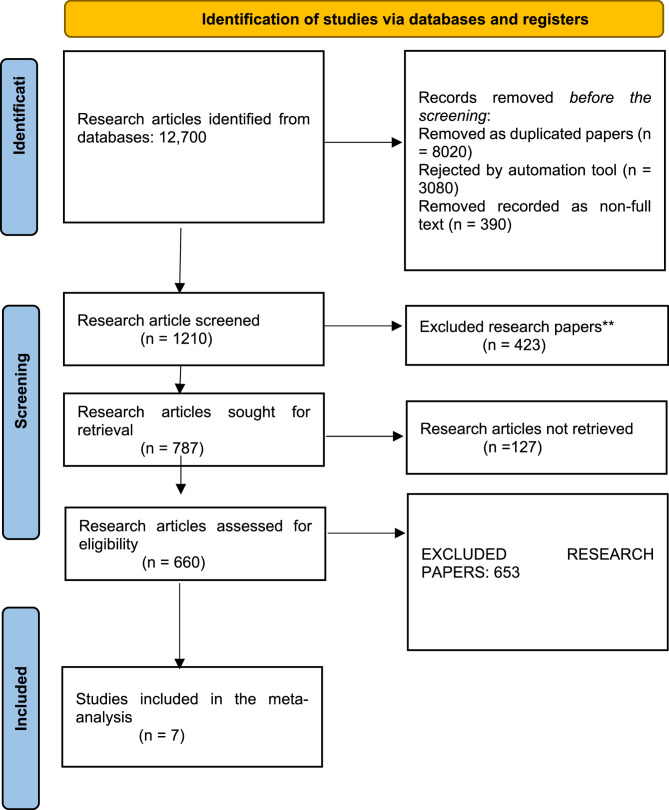
PRISMA Flowchart for screening and selection of included studies

The selection and screening of research articles on title “*Comparative Effectiveness of Individual GLP-1 Receptor Agonists on Colorectal Cancer Risk*” was performed by following the PRISMA guidelines in this meta-analysis. A previously discussed search approach was used to extract 12,700 research articles. Before the final screening, 787 articles were retrieved from the initial screening of only 1210 publications. Only 660 were evaluated for eligibility criteria, and seven research publications remained when the exclusion criteria were applied, as illustrated in Fig. 1.

### Characteristics of included studies

This meta-analysis analyzed seven retrospective cohort studies and 5,066,681 patients receiving GLP-1 RAs to evaluate their effects on colorectal cancer risk and incidence. The main characteristics of the studies selected for the pooled analysis are presented in Table 1. The sample size for the included studies ranged from 16,446 to 1,651,452 patients receiving GLP-1. The follow-up period ranged from six to 15 years. Most of the included studies compared the glucagon-like peptide-1 (GLP-1) receptor agonists with insulin and metformin. The included cohorts were from the USA [33–35], Canada [23, 25, 36] and China [37].

**Table 1:** Characteristics of included studies

Author, year	Country	Study Population (mean age)	Study Design	Type of treatment	Study groups	Study follow up	Incidence rates	Unadjusted HR
Htoo et al., 2016 [33]	USA	60,366 patients (71.8 years)	Cohort study	glucagon-like peptide-1 (GLP-1) receptor agonists versus long acting insulin (LAI)	GLP-1ra: 5600 LAI: 54,767	6 months	**T**: 11 **C**: 276	T: 0.82 (0.42–1.58)
Wang et al., 2024 [38]	Canada	1,651,452 patients with T2D (59.8 years)	Retrospective cohort study	glucagon-like peptide-1 (GLP-1) receptor agonists versus insulin	GLP-1: 48 983 Insulin: 1 044 475	1.5 years	T: 223 C: 391	0.54 (0.46–0.64)
Wang et al., 2022 [34]	USA	683,570 patients (60 years)	Cohort study	glucagon-like peptide-1 (GLP-1) receptor agonists versus metformin	GLP-1: 64 230 Metformin: 619 340	1 year		0.82 (0.65–0.91)
Wang et al., 2024 [23]	Canada	1 221 218 drug-naive patients with T2D (59.8 years)	Cohort study	glucagon-like peptide-1 (GLP-1) receptor agonists versus metformin		15 years		0.56 (0.44–0.72)
Hsieh et al., 2024 [35]	USA	16,446 female with PCOS (41.8 years)	retrospective cohort study	glucagon-like peptide-1 (GLP-1) receptor agonists versus metformin		15 years		0.35 (0.22–0.57)
Sun et al., 2024 [37]	China	933,970 patients (47.9 years)	retrospective cohort study		80 154 cases and 853 816 controls			1.12 (1.07–1.18)
Abrahami et al., 2018 [36]	Canada	500,659 patients (55.6 years)	Cohort study	glucagon-like peptide-1 (GLP-1) receptor agonists versus DPP-4 inhibitors	GLP1: 388,619 DPP-4: 112,040	1 year	T: 733	1.0 (0.22–1.6)

### Quality assessment of included studies

Among the seven included studies, four were at low risk [33–36], and three had moderate risk [23, 25, 37] as represented in Table 2. Most comparisons showed low to moderate evidence quality, and the study’s limitations, inconsistencies, indirectness, and imprecision were the key reasons for the decline in confidence.

**Table 2:** Quality assessment of included studies by Newcastle-Ottawa scale

Study	Selection				Comparability		Outcome			
	Representative of the exposed cohort	Selection of external control	Ascertainment of exposure	Outcome of interest not present	Main factor	Additional factor	Assessment of outcome	Sufficient follow up time	Adequacy of follow up time	Total scores
Htoo et al., 2016 [33]	*	0	*	0	*	0	*	*	*	6/9
Wang et al., 2024 [38]	*	*	*	0	*	0	*	0	0	5/9
Wang et al., 2022 [34]	*	*	0	*	*	*	*	0	*	7/9
Wang et al., 2024 [23]	*	*	0	*	*	0	*	0	0	5/9
Hsieh et al., 2024 [35]	*	0	*	0	*	*	*	*	*	7/9
Sun et al., 2024 [37]	*	*	*	0	*	0	*	0	0	5/9
Abrahami et al., 2018 [36]	*	*	0	*	*	*	*	8	*	8/9

### Primary outcomes

#### Risk of colorectal cancer

**Fig. 2:**
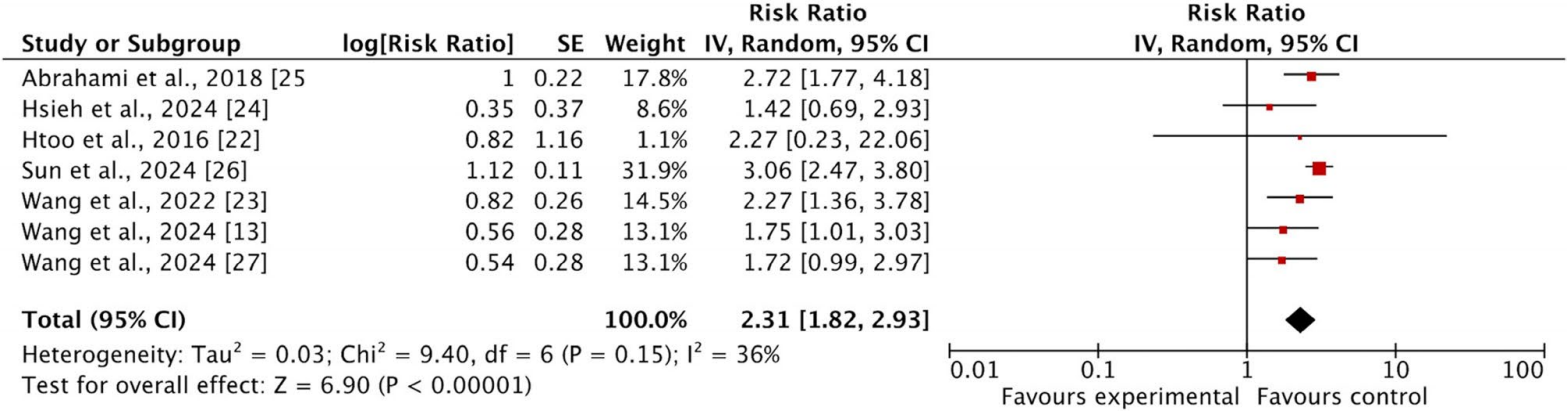
Forest plot of risk ratio of colorectal cancer among patients receiving GLP-1 RAs

**Fig. 3:**
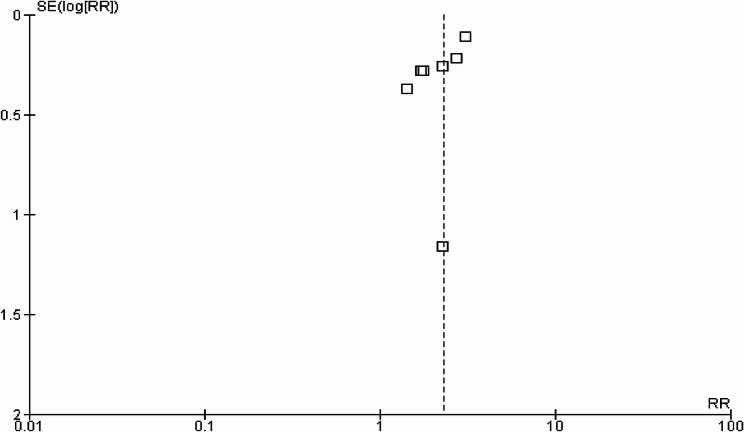
Funnel plot of risk ratio of colorectal cancer among patients receiving GLP-1 RAs

All seven included studies discussed the colorectal cancer risk as an outcome among patients receiving GLP-1 RAs. The pooled analysis revealed a significant increase in the risk of colorectal cancer among patients receiving GLP-1 RAs (RR: 2.31 [1.82–2.93], I2 = 36%, *p* < 0.0001), as illustrated in Fig. 2. The funnel plot showed a symmetrical distribution of studies that reported low publication bias (Fig. 3).

Publication bias of the included studies suggested a large symmetry whose interpretation is inherently limited due to the small number of studies and was assessed using funnel plots, highlighting the need for the implementation of other formal statistical tests for funnel plot asymmetry, such as Egger’s test or Begg’s test. Although these tests require a large number of studies for reliable inference, formal statistical tests for the assessment of publication bias were not performed, as the results would be unreliable and potentially misleading.

#### Incidence of colorectal cancer

**Fig. 4:**
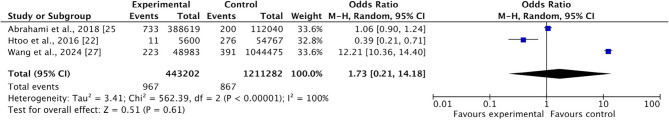
Forest plot of odd ratio of incidence of colorectal cancer associated with GLP-1 RAs

Among the seven included studies, three reported the incidence rates of colorectal cancer as outcomes among patients receiving GLP-1 RAs compared to other drugs such as DPP-4 or metformin. The pooled analysis revealed that the incidence of colorectal cancer was not significantly associated with GLP-1 RAs compared to other drugs (OR: 1.73 [0.21–14.18], *p* = 0.61, I2 = 100%), as shown in Fig. 4.

## Discussion

This study aimed to assess the association between individual GLP-1 receptor agonists and Colorectal Cancer risk among patients by adopting a meta-analysis approach. Through the analysis of seven retrospective cohort studies involving 5,066,681 patients who received GLP-1 RAs, the findings reported that colorectal cancer risk was significantly associated with the use of GLP-1 RAs. The pooled analysis reported that the risk of colorectal cancer increased dramatically among patients receiving GLP-1 RAs [RR: 2.31 (1.82–2.93) I2 = 36%, *p* < 0.0001], suggesting a more than two-fold increase in risk, while the incidence of colorectal cancer was not significantly associated with GLP-1 RAs compared to other drugs [OR: 1.73 (0.21–14.18), *p* = 0.61, I2 = 100%]. Among the seven included studies, four were at low risk [33–36], and three had a moderate risk [23, 25, 37]. I^2^ scores ranged from 36 to 100%, indicating substantial heterogeneity across the included studies. The funnel plot showed a symmetrical distribution, indicating low publication bias and increased confidence in the reliability of the results.

These findings were inconsistent with those of previous studies, which reported a strong association between GLP-1 RAs and various types of cancers. Figlioli et al. [39] analyzed the uncertain association between glucagon-like peptide-1 receptor agonists and risk of gastrointestinal cancer. These findings reported no significant impact of GLP-1 RAs on the risk of gastrointestinal cancer. Previous studies of the association between GLP-1 RAs and cancer risk have reported inconsistent results. The proproliferative effects of GLP-1 RAs on gastrointestinal tissues have been questioned in preclinical studies, although their anti-inflammatory and metabolic properties suggest a protective role [40]. Mechanisms such as accelerated cell turnover or altered gut microbiota composition caused by GLP-1 RA use may be part of the elevated risk observed in the context of colorectal cancer. However, it is unknown whether GLP-1 RAs are the sole cause of the elevated risk or whether additional confounding variables are at play, such as pre-existing metabolic disorders, insulin resistance, or chronic diabetes [41]. Similarly, Liu et al. [42] conducted a meta-analysis to evaluate the risk of malignant neoplasia after GLP-1 receptor agonist administration and reported no increase in the risk of malignant neoplasia among individuals with type 2 diabetes who received GLP-1 receptor agonist administration. Cao et al. [28] analyzed the risk of cancers associated with GLP-1RAs in patients with T2DM. The findings showed reductions in the overall risk of cancer after the use of GLP-1 RAs.

The findings of this study suggest complex associations between the use of GLP-1 RAs and colorectal cancer risk. The findings reported an increased risk of colorectal cancer, with non-significant results for colorectal cancer incidence. This difference in findings revealed that colorectal cancer risk and incidence were not directly interconnected [43]. In this analysis, the increased risk was aligned with concerns discussed in earlier cohort studies. It have been demonstrated that the GLP-1 RAs cause hyperplasia in specific gastrointestinal tissues. It is crucial to consider the research design and follow-up period when evaluating the results because large-scale clinical trials, such as the LEADER trial, have not continuously indicated an increased cancer risk [44]. Populations receiving GLP-1 RAs may be at a higher risk of colorectal cancer due to multiple factors, such as insulin resistance, chronic inflammation, and obesity. These factors are strongly linked with colorectal cancer and may prove the strong association between GLP-1 RAs and cancer risk. Furthermore, the findings related to colorectal cancer incidence illustrate the issue of rare outcomes among clinical trials, owing to the low incidence rates of colorectal cancer over shorter follow-up periods [45].

The findings of this study have several important clinical implications. Given the observed increased risk of colorectal cancer among patients receiving GLP-1 receptor agonists, clinicians should approach long-term prescribing of these agents with careful consideration, particularly in individuals with additional risk factors for colorectal cancer, such as a family history, obesity, inflammatory bowel disease, or long-standing type 2 diabetes. Although GLP-1 RAs are frequently recommended due to their cardiometabolic advantages, careful observation is necessary due to the possibility of increased gastrointestinal tissue proliferation and the corresponding risk of cancer. When starting GLP-1 RAs, these findings suggest the need for individualized medicine strategies, which may include careful patient selection, risk-benefit analyses, and possibly introducing earlier or more frequent colorectal cancer screening for high-risk patients. Crucially, the discrepancy between cancer incidence and risk shows that while the discovered correlations might not immediately result in a significant clinical burden of disease, they should not be discounted in the absence of additional long-term safety information. Clinicians should exercise caution until larger, carefully planned prospective trials elucidate these correlations, particularly when administering GLP-1 RAs for prolonged periods of time in individuals that are already at risk for colorectal cancers.

This study has several limitations, but also offers numerous advantages. First, the number of included studies on colorectal cancer risk is limited. Further studies are needed to enhance the validity and reliability of this meta-analysis. Second, the high heterogeneity of cancer incidence suggests significant differences in study populations, study methods, and outcomes. Furthermore, the cohort nature of the included studies increased the potential for confounding bias, which may have influenced the findings. Due to the involvement of similar population groups and drug types, we cannot conduct a subgroup analysis to reduce heterogeneity. Additionally, all included studies have not specified the kind of GLP-1 RA involved in boosting colorectal cancer risk, so the subgroup analysis could not be conducted based on drug type. Third, significant variations in the duration of follow-up of the included studies may have impacted the findings related to colorectal cancer risk. The disparity between the risk and incidence results might also reflect the difficulties in researching cancer outcomes in communities with low overall incidence, where conclusive results necessitate large sample numbers and long-term data.

Although a standard random-effects model was employed to account for between-study variability, alternative methodological approaches could have further enhanced the robustness of this analysis. Fourth, the funnel plot, based on only seven studies, may not be reliable for detecting such publication bias. Despite the visual appearance of a funnel plot, the small number of included studies significantly restricts the ability to rule out publication bias definitively, and the implementation of other statistical tests for asymmetry is underpowered with few studies and unreliable results. There is also a possibility that the included studies reporting null or negative associations may be underrepresented in the published literature, leading to an overestimation of the observed association between GLP-1 RAs and CRC susceptibility. Thus, the interpretation of publication bias is another limitation of this study. Alternative statistical methods, such as Egger’s regression test, offer more objective measures of publication bias; however, they were not applied here due to the limited number of included studies, which could reduce their statistical power and accuracy. Lastly, the number of studies was limited to predict the outcome related to the incidence of colorectal cancer associated with GLP-1 RA use. The insufficient availability of data in the included studies results in a lack of granularity, which is basically required to perform subgroup analysis for specific GLP-1 RAs (e.g. liraglutide, semaglutide, or exenutide). While the implementation of subgroup analysis based on individual SLP-1 RAs appears to significantly enhance the clinical acceptability of these findings by differentiating the potential risk profiles of individual drugs, it seems to be involved in a significant enhancement of the clinical acceptability of these findings. This reflect that the effect of GLP-1 RA as a class and clinicians should interpret these findings to ensure that the future studies must comprehend detailed information to investigate the potential differences in CRC risk with individual GLP-1 RAs.

## Conclusion

Overall, the findings showed a significantly increased risk of CRC among patients receiving GLP-1 RAs. However, the incidence of colorectal cancer was not significantly associated with GLP-1 RA use. These findings require further investigation regarding the long-term safety of GLP-1 RAs in populations receiving GLP-1 RAs. Further long-term clinical trial-based research should be conducted on a larger scale to highlight the potential relationship between GLP-1 RAs and cancer risk in various populations.

## Supplementary Information

Supplementary Material 1.

## Data Availability

The manuscript includes all necessary data, and related data may be provided upon request from the corresponding author/s.
