# Supplementary material for: Association between GLP-1 receptor agonists as a class and colorectal cancer risk: a meta-analysis of retrospective cohort studies
Source: BMC Gastroenterol. 2025 Aug 22;25:614. doi: 10.1186/s12876-025-04211-4 (PMC12372225; doi:10.1186/s12876-025-04211-4)
Supplement: Supplementary file 1 — Supplementary Material 1. [file 12876_2025_4211_MOESM1_ESM.docx]

**Supplementary Table**

| **Section** | **Sub-item** | **Star** |
| --- | --- | --- |
| Selection | 1. Representativeness of exposed cohort | ★ |
|  | 2. Selection of non-exposed cohort | ★ |
|  | 3. Ascertainment of exposure | ★ |
|  | 4. Outcome not present at start | ★ |
| Comparability | 1. Control for important factors 2. Additional factor | ★  ★ |
| Outcome | 1. Assessment of outcome | ★ |
|  | 2. Sufficient follow-up duration | ★ |
|  | 3. Adequacy of follow-up | ★ |
| **Total Stars** |  | **9/9** |
